# Second cancer risk following Hodgkin lymphoma

**DOI:** 10.18632/oncotarget.20876

**Published:** 2017-09-14

**Authors:** Amit Sud, Kari Hemminki, Richard S. Houlston

**Affiliations:** Amit Sud: Division of Genetics and Epidemiology, The Institute of Cancer Research, London, UK

**Keywords:** Hodgkin lymphoma, second cancer risk, family history

**Figure 1:**
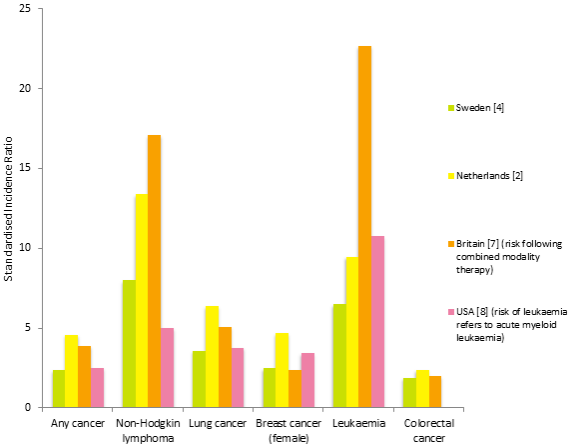
Second cancer in HL survivors in different studies [2, 4, 7, 8] Risk estimates from the British cohort calculated from HL patients treated with combined modality therapy whereas other cohorts include any treatment modality. Risk estimate for leukaemia in USA, refers to acute myeloid leukaemia. No risk estimate for colorectal cancer in HL survivors for USA study.

Whilst the development of chemotherapy and radiotherapy for Hodgkin lymphoma (HL) has resulted in high cure rates, long-term side effects of treatment including cardiovascular disease and second cancer represent a significant cause of morbidity and mortality for HL survivors. Since an increased risk of second cancer was first observed in HL survivors in the 1970s, progress has been made in quantifying this cancer risk (Figure 1), as well as identifying patient and treatment related factors influencing risk. These factors include age at treatment, site and dose of radiotherapy and chemotherapy, and smoking. Knowledge of second cancer risks has informed recent HL treatment strategies, namely the development of treatment regimens based on a reduction in the field and dose of radiotherapy and chemotherapy and risk-adapted therapy [1]. Despite such modifications, a recent analysis of a cohort of HL survivors in the Netherlands failed to demonstrate a reduction in second cancer risks in HL survivors treated in the 1990s when compared to earlier periods [2].

Approximately 20 years ago a family history of breast cancer was suggested as a possible influence on second cancer risk following HL [3]. Subsequent studies have attempted to quantify the impact of family history on second cancer risk in HL survivors, but have had limited power. Using a Swedish Family Cancer Project Database, we sought to gain insight into the risk of second cancer after a diagnosis of HL and its relationship to temporal changes in treatment regimens. Furthermore, we performed a detailed analysis of the influence of family history, as a surrogate for genetic susceptibility, on the risk of second cancer in patients with HL [4].

We identified 9,522 HL patients in Sweden from 1965-2012, with a total of 1,215 second cancers observed in 1,121 patients. The risk of all second cancers was elevated after HL diagnosis, with a standardised incidence ratio (SIR) of 2.39, corresponding to an absolute excess (AER) risk of 71.2 cases per 10,000 person-years. Non-Hodgkin lymphoma, breast cancer, non-melanoma skin cancer, leukaemia and colorectal cancer contributed the most to AERs, accounting for 16.2%, 14.5%, 12.9%, 11.4%, 9.7% and 7.6% of the excess cancer risks respectively. We confirmed the findings of previous studies demonstrating distinct differences in second cancers risk depending on cancer site, sex and age at HL treatment.

Given an absence of individual treatment data in the Swedish Cancer Registry and that treatment principles for HL in Sweden are broadly similar to those of other Western countries, we analysed second cancer risk for HL patients diagnosed in the time periods of 1965 to 1977, 1978 to 1988, and 1989 to 2000 as a proxy for treatment strategy. We found no evidence for significant change in the risk of second cancer, supporting the recent findings of Schaapveld *et al* [2]*.* Possible explanations for this finding include an increase in the proportion of patients exposed to chemotherapy in later time periods, the impact of screening, an interaction between less toxic chemotherapy and second cancer risk, and the time periods analysed not accurately capturing the reduction in treatment intensity.

We also found an increase in second cancer risk in HL survivors who had a first-degree relative (FDR) with cancer. This elevated risk was correlated with the number of FDRs affected with cancer, with respective SIRs being 2.67 and 3.40 for patients with one and two or more affected FDRs. For lung, colorectal and breast cancer, we observed a 3.3-fold, 2.1-fold, and 1.8-fold increase in risk in HL survivors with a FDR with the respective site-specific cancer. It was notable that a greater than additive interaction between family history of lung cancer and HL treatment was observed, which may be explained by environmental factors such as smoking. These findings provide indirect evidence for a role in genetic predisposition in determining second cancer risk and are supported by the example of retinoblastoma, in which individuals with hereditary retinoblastoma have a much higher risk of radiotherapy-induced second malignancy compared with those with sporadic disease [5]. Whilst no high impact cancer susceptibility gene has been identified in HL, polygenic susceptibility may provide an alternative model by which germline genetics influence second cancer risk [6].

Our study further substantiates the significant cancer risks associated with survivorship from HL and that these risks are modified by a number of factors including family history of cancer. Understanding the biological mechanisms of such associations will further our knowledge of carcinogenesis, in the hope of developing novel therapeutic strategies. Moreover, such information has current direct clinical relevance in planning risk-adapted therapy, implementing strategies to reduce second cancer risk such as smoking cessation and informing the screening of HL survivors.
